# Patient, physician, encounter, and billing characteristics predict the accuracy of syndromic surveillance case definitions

**DOI:** 10.1186/1471-2458-12-166

**Published:** 2012-03-08

**Authors:** Geneviève Cadieux, David L Buckeridge, André Jacques, Michael Libman, Nandini Dendukuri, Robyn Tamblyn

**Affiliations:** 1 Department of Epidemiology and Biostatistics, McGill University, 1020 Pine Avenue West, Montreal, QC, H3A 1A2, Canada; 2 Direction de la Santé Publique de Montréal, 1301 Sherbrooke Street East, Montreal, QC, H2L 1M3, Canada; 3 Collège des Médecins du Québec, 2170 René-Lévesque Boulevard West, Montreal, QC, H3H 2T8, Canada; 4 Department of Medicine, McGill University, 3655 Sir William Osler Promenade, Montreal, QC, H3G 1Y6, Canada

## Abstract

**Background:**

Syndromic surveillance systems are plagued by high false-positive rates. In chronic disease monitoring, investigators have identified several factors that predict the accuracy of case definitions based on diagnoses in administrative data, and some have even incorporated these predictors into novel case detection methods, resulting in a significant improvement in case definition accuracy. Based on findings from these studies, we sought to identify physician, patient, encounter, and billing characteristics associated with the positive predictive value (PPV) of case definitions for 5 syndromes (fever, gastrointestinal, neurological, rash, and respiratory (including influenza-like illness)).

**Methods:**

The study sample comprised 4,330 syndrome-positive visits from the claims of 1,098 randomly-selected physicians working in Quebec, Canada in 2005-2007. For each visit, physician-facilitated chart review was used to assess whether the same syndrome was present in the medical chart (gold standard). We used multivariate logistic regression analyses to estimate the association between claim-chart agreement about the presence of a syndrome and physician, patient, encounter, and billing characteristics.

**Results:**

The likelihood of the medical chart agreeing with the physician claim about the presence of a syndrome was higher when the treating physician had billed many visits for the same syndrome recently (OR_per 10 visit_, 1.05; 95% CI, 1.01-1.08), had a lower workload (OR_per 10 claims_, 0.93; 95% CI, 0.90-0.97), and when the patient was younger (OR_per 5 years of age_, 0.96; 95% CI, 0.94-0.97), and less socially deprived (OR_most versus least deprived_, 0.76; 95% CI, 0.60-0.95).

**Conclusions:**

Many physician, patient, encounter, and billing characteristics associated with the PPV of surveillance case definition are accessible to public health, and could be used to reduce false-positive alerts by surveillance systems, either by focusing on the data most likely to be accurate, or by adjusting the observed data for known biases in diagnosis reporting and performing surveillance using the adjusted values.

## Background

Syndromic surveillance systems were adopted promptly in the wake of 9/11 amidst concerns of bioterrorism; their primary purpose was to detect disease outbreaks and bioterrorism events rapidly. To ensure that no outbreak would be missed, syndromic surveillance systems were initially designed to alert at very low thresholds. As a consequence of this design, syndromic surveillance systems' usefulness for public health has been stymied by high rates of false-alerts [1,2]. Few have attempted to improve the accuracy of syndromic surveillance systems, and they have done so either by modifying statistical outbreak detection algorithms [3-5] or by using different data sources [6,7].

In contrast, in chronic disease monitoring, investigators have identified several factors that predict the accuracy of case definitions based on diagnoses in administrative data [8-16]. These studies have enabled a new generation of advanced methods for disease surveillance to be created that incorporate these predictors into novel case detection methods. As a result, there has been significant improvement in case definition accuracy in chronic disease [17-20]. Specifically, these investigators found that characteristics of the physician (e.g., workload [21]), patient (e.g., comorbidity [8,9,22]), encounter (e.g., emergency admission [11]), and healthcare site (e.g., hospital volume [12]) were associated with the accuracy of case definitions based on administrative data (see Additional file 1 for summary review). Similar factors may influence the accuracy of syndromic surveillance case definitions. However, to date, no one has attempted to identify predictors of syndromic surveillance case definition accuracy. Building on the findings from chronic disease monitoring, we anticipate that the following physician, patient, encounter, and billing characteristics may be predictive of the accuracy of syndromic surveillance case definitions based on administrative data.

### Hypothesized predictors of the accuracy of syndromic surveillance case definitions based on diagnoses in administrative data

#### Physician characteristics

Greater experience appears to be associated with lower accuracy of billing diagnosis [21] and diagnostic coding [23], perhaps because more experienced physicians are less likely to use reference materials to inform their coding [23]. As compared to generalists, specialists see a narrower segment of the patient population for a subset of health conditions; they likely use fewer diagnostic codes and may therefore have better billing diagnosis accuracy. Several other physician characteristics may also be relevant; physician gender and language are associated with several practice style indicators, including physician-patient communication [24-27], and may be associated with billing diagnosis accuracy.

#### Patient characteristics

Treating more complex patients likely requires more working memory and increases physician cognitive load [28], and thus greater patient complexity may negatively affect billing diagnosis accuracy. Indicators of patient complexity including age [8,10,12,14,20,22,29], comorbidity [8,9,30], socioeconomic status [22,29], and health services utilization [9,10] have been shown to impact the accuracy of case definitions for chronic diseases. Patient gender [8] has also been shown to influence the accuracy of case definitions, perhaps through patient-physician communication [24-27].

#### Encounter characteristics

The context for the clinical encounter influences how much time and what resources are available for billing, and likely impacts diagnostic data accuracy. Prior studies suggest that physician errors in diagnosis vary by type of health condition treated [15], healthcare site [15], and physician workload [21]. Weekend medical encounter are more likely to be limited to a specific acute or urgent health complaint; therefore, diagnoses for those encounters may be more accurate, particularly for infectious disease. Similarly, encounters may be more focused and claim diagnosis accuracy may be better when the physician is not/less familiar with the patient. Previous studies have shown that rare diagnoses in administrative data are more likely to be erroneous than common ones [31,32]; therefore, health conditions encountered often by physician are expected to be recorded more accurately in claims. Through a similar mechanism, syndromes that undergo seasonal variation are expected to be more accurately recorded in claims during 'peak season'.

#### Billing practices

No prior study has examined the relationship between billing practices and billing diagnosis accuracy; however, several attributes of billing practices likely influence the accuracy of diagnoses in administrative data. Accuracy is expected to be higher when the treating physician does the billing, as compared to clerical staff, because the treating physician has detailed knowledge of the case. Billing diagnoses that are automatically abstracted from the 'reason for visit' field of an electronic medical record are expected to be more accurate than billing diagnoses resulting from parallel manual data entry for billing purposes. Billing volume may also be associated with diagnostic coding accuracy. The proportion of billed visits with missing or unspecified diagnoses likely reflects attention to diagnostic coding, and may be associated with accuracy of diagnoses in administrative data. The breadth of diagnostic codes used by a physician likely reflects the scope of health conditions treated; a narrower scope of practice may be associated with more accurate diagnostic coding.

Whereas some of the previously identified predictors of case definition accuracy may be specific to certain chronic diseases (e.g., older age being associated with better case definition accuracy for chronic diseases that are more prevalent among older adults, such as dementia [29], osteoporosis [17], and osteoarthritis [20]) these studies provide clues as to what types of factors may predict the accuracy of syndromic surveillance case definitions. The objective of the present study was to evaluate whether or not the aforementioned physician, patient, encounter, and billing characteristics are associated with the positive predictive value (PPV) of syndromic surveillance case definitions based on diagnoses in physician claims.

## Methods

### Context

This study was conducted in the province of Quebec, Canada, where universal health coverage is provided through the provincial health insurance plan. Similar to health maintenance organizations and medical provider networks, each Canadian province maintains a population-based registry of insured persons and claims for all physician visits remunerated on a fee-for-service basis. The registrant database includes patient first and last name, sex, date of birth, unique lifelong medical insurance identifier, and 6-digit postal code, the latter enabling linkage to census information by geographic area of residence. Physician claims include information on the principal diagnosis for the visit (one diagnosis per claim), medical procedure, visit date, and clinic type and location. All claims also record unique physician and patient identifiers that can be used to create longitudinal histories of healthcare use. In the province of Quebec, 99% of residents have provincial health insurance, and 85-95% of medical visits are remunerated on a fee-for-service basis [33].

### Study design and population

In a prior study assessing the accuracy of syndrome definitions based on diagnoses in physician claims [31], we randomly selected a cohort of 3,600 physicians who were practicing in the fee-for-service system in the province of Quebec in 2005-2007, and who were likely to provide first-contact care. In 2005-2007, these 3,600 study physicians billed for over 20 million visits by 4.8 million patients (61% of the province's population) from their community-based practices. For each physician, we selected a stratified random sample of 5 visits with a syndrome-positive diagnosis in the claim, i.e., 1 visit for each of 5 syndromes of public health importance [34]: fever, gastrointestinal, neurological, rash, and respiratory, including influenza-like illness (ILI), large-group definition [35]. The present study is based on the cohort of 1,098 physicians (participation rate of 33.7%) who were eligible and consented to provide medical chart information, and 4,330 of their visits with a syndrome-positive claim diagnosis [31].

### Outcome measure

For each visit with a syndrome-positive diagnosis in the physician claim, we assessed whether the same syndrome was documented in the medical chart. Medical chart data was retrieved using a previously described physician-facilitated chart review methodology [36].

### Potential predictors of the accuracy of syndromic surveillance case definitions based on physician claim diagnoses

#### Physician characteristics

*Physician gender *and *language *(French or English) were obtained from the provincial health insurance agency. *Years since licensure *was calculated by subtracting the year of licensure, which was obtained from the provincial medical regulatory authority, from the year of the syndrome-positive visit. *Physician specialty *was obtained from the provincial health insurance agency.

#### Patient characteristics

*Patient gender *was obtained from the registrant database. *Patient age *on October 1^st ^of the study year when the visit took place was obtained from the provincial health services agency. For each patient, complexity was assessed by the *Charlson Comorbidity Index *[37], which was computed using diagnoses in claims billed by all physicians seen during the year preceding the visit, as well as the *number of ambulatory care visits *in the previous year. *Material and social deprivation indices*, developed by the Quebec National Public Health Institute [38,39], were calculated for each patient using Statistics Canada's 2006 census data. The material deprivation index summarizes information on the proportion of persons who have no high school diploma, the proportion of persons employed, and the average income in the patient's postal code area of residence. The social deprivation index summarizes information on the proportion of single-parent families, the proportion of persons living alone, and the proportion of persons separated, divorced, or widowed in the patient's 6-digit postal code area of residence.

#### Encounter characteristics

*Syndrome type *(fever, gastrointestinal, neurological, rash, and respiratory including ILI) was derived from the from physician claim diagnosis. The *type of clinic *was obtained from the physician claim and categorized as private clinic, community health center, or hospital ambulatory care clinic. The *geographic location of the clinic *was categorized as urban or rural based on the clinic's postal code. The *day of the week *and *season *during which the encounter took place was derived from the encounter date in the physician claim. As an indicator of syndrome frequency, the *number of visits for the same syndrome billed by the study physician in the previous 30 days *was calculated from each physician's claims. *Physician workload *was calculated as the number of physician claims on the day of the encounter, which reflects both the number of patients seen and the complexity of their care. *Physician familiarity with the patient *was assessed by determining whether or not the physician had treated the patient in the previous year.

#### Billing practices

The type of *billing software *used and *what person entered the diagnostic code in the claim *were obtained through a telephone interview with the physician [31]. Physicians' *annual billing volume *was calculated as the number of distinct claims billed by a physician during the study year when the syndrome-positive visit occurred. The *percent of visits with a missing or unspecified diagnostic code *was calculated as the total number of visits without any diagnostic code or with a diagnostic code of 'V999' (unspecified), divided by the total number of visits billed by the physician during the study year, multiplied by 100. The *number of distinct diagnostic codes used *was calculated as the number of distinct diagnostic codes used among all claims billed during the study year when the syndrome-positive visit occurred.

### Statistical methods

Multivariable logistic regression analyses for clustered data were performed using generalized estimating equations (GEE) to estimate the association between the presence or absence of the syndrome in the medical chart (binary dependent variable) for a given visit with a syndrome-positive diagnosis in the physician claim, and physician characteristics, billing practices, patient characteristics, and encounter characteristics (SAS Version 9.2, SAS Institute Inc., Cary, NC). The visit was the unit of analysis, and visits were clustered within study physicians (there was only 1 visit per patient). Based on the assumption that physician diagnostic coding and billing patterns may change over time, visits were ordered chronologically, and a first-order autoregressive correlation structure of residuals was used to account for clustering. A 2-sided test with a p-value of 0.05 was used to assess statistical significance. In the main analyses, physician time since licensure, billing characteristics, and patient age, health services utilization, and Charlson comorbidity index were modelled as continuous variables, assuming the linearity of their association with the logit of the probability of the presence or absence of the syndrome in the medical chart. In sensitivity analyses, to account for possibly non-linear relationships, continuous variables were categorized into quartiles and modelled through three dummy indicators with the lowest quartile as the reference; we also tested the statistical significance of the quadratic component.

### Ethics review

The research protocol for this study was reviewed and approved by the McGill University Institutional Review Board, the Quebec privacy commission, the Quebec health insurance agency, and the Quebec medical regulatory authority.

## Results

Of 4,330 visits with a syndrome-positive diagnosis in the physician claim, 2,967 (68.5%) visits accurately represented the primary reason for the visit when compared to the medical chart.

### Physician characteristics

The PPV of syndrome definitions based on physician claim diagnoses decreased by 4% with every 5 additional years since medical licensure (OR_per 5 years _, 0.96; 95% CI, 0.92-1.00) (Table 1). As compared to general practitioners, internists and general surgeons had 41% poorer PPV (OR, 0.59; 95% CI, 0.35-0.98). Physician gender and language were not significantly associated with the PPV of syndrome definitions based on physician claim diagnoses.

**Table 1:** Physician characteristics associated with accuracy of syndrome definitions based on physician claims (OR >1.00 means the encounter characteristic increased the PPV of the syndrome definition, OR < 1.00 means the encounter characteristic reduced the PPV)

	No. visits with a syndrome-positive physician claim						Bivariate regression analysis			**Multivariate regression analysis**^1^		
**Physician characteristics**	**Syndrome-positive in the chart** **(N = 2,967)**		**Syndrome-negative in the chart** **(N = 1,363)**		**Total** **(N = 4,330)**		**OR**	**95% CI**	**P value**	**OR**	**95% CI**	**P value**

	**No**.	**%**	**No**.	**%**	**No**.	**%**						

Gender:												

Female	1,164	39.2	523	38.4	1,687	39.0	Ref.	Ref.	Ref.	Ref.	Ref.	Ref.

Male	1,803	60.8	840	61.6	2,643	61.0	0.97	(0.83, 1.12)	0.64	1.13	(0.96, 1.33)	0.13

Preferred language:												

French	2,743	92.5	1,253	91.9	3,996	92.3	Ref.	Ref.	Ref.	Ref.	Ref.	Ref.

English	224	7.5	110	8.1	334	7.7	0.93	(0.69, 1.25)	0.63	0.94	(0.69, 1.26)	0.66

Specialty:												

General practice	2,721	91.7	1,246	91.4	3,967	91.6	Ref.	Ref.	Ref.	Ref.	Ref.	Ref.

Pediatrics	203	6.8	75	5.5	278	6.4	1.24	(0.88, 1.77)	0.22	0.83	(0.57, 1.20)	0.33

Internal medicine or general surgery	43	1.5	42	3.1	85	2.0	0.46	(0.31, 0.69)	< 0.001	0.59	(0.35, 0.98)	0.04

	**Mean**	**SD**	**Mean**	**SD**	**Mean**	**SD**						

Years since licensure (per 5 years)	22.9	9.2	23.7	9.6	23.1	9.4	0.95	(0.92, 0.99)	0.02	0.96	(0.92, 1.00)	0.04

^1 ^Multivariate analysis adjusted for all physician characteristics in Table 1, all patient characteristics in Table 2, and all encounter characteristics in Table 3.

### Patient characteristics

The PPV of syndrome definitions based on physician claim diagnoses was much poorer for older patients than for younger ones, with the PPV decreasing by 4% with every additional 5 years of patient age (OR_per 5 years_, 0.96; 95% CI, 0.94-0.97) (Table 2). Whereas patient comorbidity was negatively associated with the PPV in bivariate analyses (OR_per 1-point increase in Charlson comorbidity index_, 0.92; 95% CI, 0.86-0.97), the association was no longer significant when the model was adjusted for patient age (OR_per 1-point increase in Charlson comorbidity index_, 0.98; 95% CI, 0.92-1.05). Similarly, health services utilization was significantly and negatively associated with the PPV (OR_per additional visit_, 0.99; 95% CI, 0.98-0.99), but the association did not remain statistically significant in multivariate analyses. The PPV of syndrome definitions was significantly lower for patients in the highest quintile of social deprivation (OR_most versus least deprived_, 0.76; 95% CI, 0.60-0.95), as compared to those in the least socially deprived quintile. The PPV of syndrome definitions was lower among patients with the most material wealth (i.e., patients in the least materially deprived quintile) as compared to patients with more material deprivation (OR_material deprivation quintile 1 versus 3_, 1.44, 95% CI, 1.15-1.81). Patient gender was not significantly associated with the PPV of syndrome definitions.

**Table 2:** Patient characteristics associated with accuracy of syndrome definitions based on physician claims (OR >1.00 means the encounter characteristic increased the PPV of the syndrome definition, OR < 1.00 means the encounter characteristic reduced the PPV)

	No. visits with a syndrome-positive physician claim						Bivariate regression analysis			Multivariate regression analysis^1^		
**Patient characteristics**	**Syndrome-positive in the chart** **(N = 2,967)**		**Syndrome-negative in the chart** **(N = 1,363)**		**Total** **(N = 4,330)**		**OR**	**95% CI**	**P value**	**OR**	**95% CI**	**P value**

	**No**.	**%**	**No**.	**%**	**No**.	**%**						
Sex:												
Female	1,810	61.0	824	60.5	2,634	60.8	Ref.	Ref.	Ref.	Ref.	Ref.	Ref.
Male	1,157	39.0	539	39.5	1,696	39.2	0.98	(0.86, 1.12)	0.75	0.89	(0.77, 1.03)	0.11
Material deprivation index:^2^												
1^st ^quintile (least deprived)	524	17.7	284	20.8	808	18.7	Ref.	Ref.	Ref.	Ref.	Ref.	Ref.
2^nd ^quintile	584	19.7	270	19.8	854	19.7	1.16	(0.94, 1.42)	0.16	1.18	(0.95, 1.46)	0.14
3^rd ^quintile	604	20.4	243	17.8	847	19.6	1.33	(1.08, 1.64)	0.01	1.44	(1.15, 1.81)	< 0.01
4^th ^quintile	581	19.6	261	19.1	842	19.4	1.21	(0.98, 1.49)	0.07	1.25	(1.01, 1.55)	0.04
5^th ^quintile (most deprived)	545	18.4	255	18.7	800	18.5	1.16	(0.94, 1.43)	0.16	1.21	(0.97, 1.50)	0.09
Social deprivation index:^2^												
1^st ^quintile (least deprived)	611	20.6	251	18.4	862	19.9	Ref.	Ref.	Ref.	Ref.	Ref.	Ref.
2^nd ^quintile	574	19.3	263	19.3	837	19.3	0.90	(0.73, 1.10)	0.30	0.91	(0.74, 1.13)	0.41
3^rd ^quintile	572	19.3	251	18.4	823	19.0	0.91	(0.74, 1.13)	0.41	0.97	(0.77, 1.21)	0.76
4^th ^quintile	554	18.7	261	19.1	815	18.8	0.87	(0.70, 1.07)	0.19	0.88	(0.70, 1.10)	0.26
5^th ^quintile (most deprived)	527	17.8	287	21.1	814	18.8	0.75	(0.61, 0.93)	0.01	0.76	(0.60, 0.95)	0.02
Deprivation indices missing:												
No	2,838	95.7	1,313	96.3	4,151	95.9	Ref.	Ref.	Ref.	Ref.	Ref.	Ref.
Yes	129	4.3	50	3.7	179	4.1	1.04	(0.73, 1.49)	0.83	1.06	(0.68, 1.64)	0.81
	**Mean**	**SD**	**Mean**	**SD**	**Mean**	**SD**						
Age (age per 5 years is used in the regression analyses)^3^	36.4	24.9	43.2	24.0	38.5	24.8	0.95	(0.93, 0.96)	< 0.0001	0.96	(0.94, 0.97)	< 0.0001
Health services utilization (no. ambulatory care visits in the previous year)^4^	9.0	10.1	10.6	12.7	9.5	11.0	0.99	(0.98, 0.99)	< 0.0001	0.99	(0.99, 1.00)	0.08
Charlson comorbidity index (per 1-point increase)^4^	0.38	0.98	0.49	1.17	0.42	1.04	0.92	(0.86, 0.97)	< 0.01	0.98	(0.92, 1.05)	0.58

^1 ^Multivariate analysis adjusted for all patient characteristics in Table 2, all physician characteristics in Table 1, and all encounter characteristics in Table 3

^2 ^The material and social deprivation indices were calculated using Statistics Canada's 2006 census data. These indices were developed by the Quebec National Public Health Institute. The material deprivation index summarizes information on the proportion of persons who have no high school diploma, the proportion of persons employed, and the average income in the patient's 6-digit postal code area of residence. The social deprivation index summarizes information on the proportion of single-parent families, the proportion of persons living alone, and the proportion of persons separated, divorced, or widowed in the patient's 6-digit postal code area of residence.

^3 ^On October 1^st ^of the study year when the visit took place. The study spanned 2 years: October 1, 2005 to September 30, 2006, and October 1, 2006 to September 30, 2007.

^4 ^Based on all medical services claims billed by all Quebec physicians (not only the 3,600 study physicians) in the year prior to the date of the syndrome-positive visit.

### Encounter characteristics

Fever syndrome had the lowest PPV of all syndromes studied; gastrointestinal syndrome (OR, 1.72; 95% CI, 1.36-2.16), neurological syndrome (OR, 1.38; 95% CI, 1.11-1.72), rash syndrome (OR, 1.89; 95% CI, 1.51-2.37), respiratory syndrome (OR, 1.66; 95% CI, 1.29-2.14), and ILI (OR, 2.68; 95% CI, 2.06-3.48), all had significantly higher PPV than fever syndrome (Table 3). With respect to physician workload, the PPV of syndrome definitions decreased by 7% with every 10 additional claims on the day of the encounter (OR_per 10 claims_, 0.93; 95% CI, 0.90-0.97). The PPV of syndrome definitions improved by 5% with every 10 visits billed by the study physician for the same syndrome in the previous month (OR_per 10 visits_, 1.05; 95% CI, 1.01-1.08). With respect to seasonality, the PPV of syndrome definitions was significantly better in spring, as compared to winter (OR, 1.29; 95% CI, 1.07-1.57); this association seemed to be strongest for fever syndrome (Additional file 2). Whereas the PPV of syndrome definitions was better for weekend visits as compared to weekday visits in the bivariate analysis (OR, 1.42, 95% CI, 1.03-1.95), this finding was not statistically significant in the multivariate analysis. Type of clinic and geographic location of the clinic were not significantly associated with the PPV of syndrome definitions based on diagnoses in physician claims.

**Table 3:** Encounter characteristics associated with accuracy of syndrome definitions based on physician claims (OR >1.00 means the encounter characteristic increased the PPV of the syndrome definition, OR < 1.00 means the encounter characteristic reduced the PPV)

	No. visits with a syndrome-positive physician claim						Bivariate regression analysis			**Multivariate regression analysis**^1^		
**Encounter characteristics**	**Syndrome-positive in the chart** **(N = 2,967)**		**Syndrome-negative in the chart** **(N = 1,363)**		**Total** **(N = 4,330)**		**OR**	**95% CI**	**P value**	**OR**	**95% CI**	**P value**

	**No**.	**%**	**No**.	**%**	**No**.	**%**						

Syndrome type:												

Fever	371	12.5	230	16.9	601	13.9	Ref.	Ref.	Ref.	Ref.	Ref.	Ref.

Gastrointestinal	572	19.3	283	20.8	855	19.8	1.57	(1.25, 1.97)	< 0.0001	1.72	(1.36, 2.16)	< 0.0001

Neurological	608	20.5	363	26.6	971	22.4	1.29	(1.05, 1.60)	0.02	1.38	(1.11, 1.72)	< 0.01

Rash	628	21.2	269	19.7	897	20.7	1.80	(1.44, 2.25)	< 0.0001	1.89	(1.51, 2.37)	< 0.0001

Respiratory	808	27.2	241	17.7	1049	24.2	1.72	(1.36, 2.17)	< 0.0001	1.66	(1.29, 2.14)	< 0.0001

ILI	555	18.7	98	7.2	653	15.1	2.98	(2.32, 3.82)	< 0.0001	2.68	(2.06, 3.48)	< 0.0001

Type of clinic:												

Private clinic	2,916	98.3	1,320	96.9	4,236	97.8	Ref.	Ref.	Ref.	Ref.	Ref.	Ref.

Community health center	10	0.3	8	0.6	18	0.4	0.58	(0.14, 2.35)	0.45	0.46	(0.11, 2.01)	0.30

Hospital-based ambulatory clinic	41	1.4	35	2.6	76	1.8	0.53	(0.30, 0.93)	0.03	0.75	(0.37, 1.53)	0.43

Geographic location of clinic:												

Urban	2,476	83.5	1,169	85.8	3,645	84.2	Ref.	Ref.	Ref.	Ref.	Ref.	Ref.

Rural	491	16.6	194	14.2	685	15.8	1.20	(0.99, 1.46)	0.07	1.19	(0.98, 1.45)	0.08

Physician familiarity with the patient (patient treated by the study physician in the previous year):												

No	1,199	40.4	475	34.9	1,674	38.7	Ref.	Ref.	Ref.	Ref.	Ref.	Ref.

Yes	1,768	59.6	888	65.1	2,656	61.3	0.79	(0.69, 0.91)	< 0.001	0.95	(0.82, 1.11)	0.53

Day of the week:												

Weekday	2,797	94.3	1,308	96.0	4,105	94.8	Ref.	Ref.	Ref.	Ref.	Ref.	Ref.

Weekend	170	5.7	55	4.0	225	5.2	1.42	(1.03, 1.95)	0.03	1.28	(0.92, 1.77)	0.15

Season:												

Winter (12/22-03/20)	737	24.8	339	24.9	1,076	24.9	Ref.	Ref.	Ref.	Ref.	Ref.	Ref.

Spring (03/21-06/20)	855	28.8	317	23.3	1,172	27.1	1.22	(1.02, 1.47)	0.03	1.29	(1.07, 1.57)	0.01

Summer (06/21-09/22)	645	21.7	351	25.8	996	23.0	0.84	(0.70, 1.01)	0.06	0.91	(0.75, 1.10)	0.33

Fall (09/23-12/21)	730	24.6	356	26.1	1,086	25.1	0.94	(0.79, 1.12)	0.48	0.97	(0.81, 1.17)	0.79

	**Mean**	**SD**	**Mean**	**SD**	**Mean**	**SD**						

No. visits for the same syndrome billed by the study physician in the previous 30 days (per 10 visits)	4.1	6.7	4.2	6.2	4.2	6.6	1.08	(0.95, 1.23)	0.25	1.05	(1.01, 1.08)	0.01

Physician workload: no. claims billed that day (per 10 claims)	35.1	17.4	36.5	21.0	35.5	18.6	0.96	(0.93, 1.00)	0.03	0.93	(0.90, 0.97)	< 0.001

^1 ^Multivariate analysis adjusted for all encounter characteristics in Table 3, all physician characteristics in Table 1, and all patient characteristics in Table 2.

### Billing practices

Several billing softwares were significantly associated with syndrome definition PPV (Table 4). *Purkinje *billing software, which abstracted the billing diagnosis from the electronic medical record in an automated manner, had a higher PPV than *Soft Informatique*, which required manual input of the billing diagnosis (OR, 1.29; 95% CI, 1.05-1.59). Surprisingly, what person entered the ICD-9 diagnostic code on the billing claim was not significantly associated with the PPV of syndrome definitions. Physician annual billing volume, proportion of visits billed with a missing or unspecified diagnostic code, and number of distinct diagnostic codes used were not significantly associated with the PPV of syndrome definitions.

**Table 4:** Billing practices associated with accuracy of syndrome definitions based on physician claims (OR >1.00 means the encounter characteristic increased the PPV of the syndrome definition, OR < 1.00 means the encounter characteristic reduced the PPV)

	No. visits with a syndrome-positive physician claim						Bivariate regression analysis			**Multivariate regression analysis**^1^		
**Billing practices**	**Syndrome-positive in the chart** **(N = 2,967)**		**Syndrome-negative in the chart** **(N = 1,363)**		**Total** **(N = 4,330)**		**OR**	**95% CI**	**P value**	**OR**	**95% CI**	**P value**

	**No**.	**%**	**No**.	**%**	**No**.	**%**						

What person entered the diagnostic code in the claim?												

Physician	443	14.9	203	14.9	646	14.9	Ref.	Ref.	Ref.	Ref.	Ref.	Ref.

Secretary or nurse	2,015	67.9	907	66.5	2,922	67.5	1.01	(0.82, 1.26)	0.91	0.93	(0.75, 1.15)	0.50

Off-site billing company or RAMQ (i.e., paper billing)^2^	509	17.2	253	18.6	762	17.6	0.92	(0.71, 1.19)	0.52	0.81	(0.62, 1.06)	0.12

Billing software used:												

Soft Informatique	715	24.4	342	25.4	1,057	24.8	Ref.	Ref.	Ref.	Ref.	Ref.	Ref.

Purkinje	721	24.7	264	19.6	985	23.1	1.30	(1.07, 1.60)	0.01	1.29	(1.05, 1.59)	0.02

ADN Medical	405	13.9	166	12.3	571	13.4	1.16	(0.90, 1.49)	0.24	1.17	(0.91, 1.50)	0.23

Omni-Med.com Caduceus	250	8.6	124	9.2	374	8.8	0.96	(0.74, 1.25)	0.77	0.94	(0.72, 1.24)	0.67

Medicus MED-WIN	123	4.2	67	5.0	190	4.5	0.87	(0.65, 1.17)	0.36	0.87	(0.64, 1.17)	0.35

Facturation.net	73	2.5	64	4.8	137	3.2	0.55	(0.35, 0.86)	0.01	0.54	(0.34, 0.85)	0.01

ANDX Xclaim	61	2.1	40	3.0	115	2.7	0.73	(0.47, 1.14)	0.17	0.70	(0.42, 1.15)	0.16

CareOffice	85	2.9	30	2.2	103	2.4	1.36	(0.84, 2.18)	0.21	1.32	(0.76, 2.27)	0.32

Médifiche	75	2.6	28	2.1	101	2.4	1.28	(0.81, 2.02)	0.29	1.24	(0.77, 1.98)	0.38

Toubib	52	1.8	43	3.2	95	2.2	0.58	(0.32, 1.05)	0.07	0.53	(0.29, 0.97)	0.04

FMP	57	2.0	16	1.2	73	1.7	1.71	(0.92, 3.19)	0.09	1.74	(0.90, 3.34)	0.10

Médicalc Inc.^3^	49	1.7	19	1.4	68	1.6	1.23	(0.61, 2.47)	0.57	1.27	(0.62, 2.62)	0.51

Param	47	1.6	18	1.3	65	1.5	1.24	(0.67, 2.29)	0.49	1.19	(0.66, 2.17)	0.56

ACL Systèmes Santé	43	1.5	20	1.5	63	1.5	1.03	(0.58, 1.84)	0.92	1.06	(0.56, 2.02)	0.85

Factura-Med	43	1.5	17	1.3	60	1.4	1.20	(0.79, 1.84)	0.39	1.24	(0.81, 1.89)	0.32

FmedX MED-Office	39	1.3	18	1.3	57	1.3	1.04	(0.48, 2.25)	0.92	0.99	(0.46, 2.13)	0.98

Sys-Thèmes	24	0.8	9	0.7	33	0.8	1.27	(0.54, 3.00)	0.59	1.24	(0.55, 2.77)	0.61

Gestimed	12	0.4	14	1.0	26	0.6	0.41	(0.21, 0.81)	0.01	0.45	(0.25, 0.84)	0.01

Salus	10	0.3	10	0.7	20	0.5	0.48	(0.18, 1.32)	0.16	0.45	(0.14, 1.44)	0.18

Logimedic	7	0.2	8	0.6	15	0.4	0.41	(0.16, 1.05)	0.06	0.39	(0.15, 1.03)	0.06

Medi-Go	2	0.1	6	0.5	8	0.2	0.16	(0.02, 1.68)	0.13	0.15	(0.01, 1.72)	0.13

Services de facturations médicales informatiques ^3^	4	0.1	3	0.2	7	0.2	0.63	(0.40, 1.01)	0.06	0.65	(0.37, 1.16)	0.14

Other^4^	13	0.4	3	0.2	16	0.4	2.12	(0.71, 6.29)	0.18	1.94	(0.71, 5.28)	0.19

Unknown	15	0.5	17	1.3	32	0.8	0.41	(0.20,0.86)	0.02	0.48	(0.24, 0.93)	0.03

RAMQ (i.e., paper billing)2	42	1.4	17	1.2	59	1.4	1.18	(0.55, 2.57)	0.67	1.39	(0.63, 3.07)	0.41

	**Mean**	**SD**	**Mean**	**SD**	**Mean**	**SD**						

Annual billing volume (per 1,000 claims)^5^	4,913	2,623	4,913	2,646	4,913	2,630	1.00	(0.97, 1.03)	0.94	1.00	(0.97, 1.04)	0.91

Percent of visits with a missing or unspecified diagnostic code^5^	2.5	5.7	2.5	5.1	2.5	5.5	1.00	(0.99, 1.02)	0.91	1.01	(0.99, 1.02)	0.34

No distinct diagnostic codes used (per 100 codes)^5^	228	88	227	97	228	91	1.01	(0.94, 1.10)	0.76	1.02	(0.92, 1.12)	0.75

1 Multivariate analysis adjusted for all billing practices in Table 4 and all physician characteristics in Table 1.

2 RAMQ: *Régie de l'assurance maladie du Québec *(provincial health agency). Few physicians submit paper billing slips (as opposed to using electronic billing software) to the provincial health agency for fee-for-service reimbursement; if they do, they are imposed a $0.50 penalty on every paper bill submitted, and a data entry clerk at the provincial health agency must enter the diagnostic code from the paper billing slip into the RAMQ's computerized billing database (this additional step is a potential source of transcription error).

^3 ^Software developed and used solely by their namesake off-site billing company.

^4 ^Single-user billing software developed by individual physicians.

^5 ^In the study year when the visit took place. The study spanned 2 years: October 1, 2005 to September 30, 2006, and October 1, 2006 to September 30, 2007.

## Discussion

We sought to identify physician, patient, encounter, and billing characteristics associated with the PPV of syndromic surveillance case definitions. Several of the predictors of syndromic surveillance case definition accuracy that we identified are readily accessible to public health departments and other organizations that routinely perform syndromic surveillance. These predictors may be used to reduce syndromic surveillance system false-positive alerts, for example, by focusing on the data most likely to be accurate or by adjusting the observed data for known biases and performing surveillance using the adjusted values; however, future research is needed to quantify the impact of our 'improved' syndrome definitions on surveillance system performance and public health practice.

Specifically, we found that visits with a syndrome-positive diagnosis in physician claims were more likely to be confirmed as syndrome-positive by the medical chart when the physician was recently licensed. This finding is similar to those of other, general studies of billing diagnosis accuracy and physician experience [21,23]. A potential explanation for this finding is that younger physicians may be more likely to give greater attention to billing; also, more experienced physicians may be more likely to 'code from memory', which has been associated with more frequent diagnostic coding errors, as compared to coding from reference materials [23]. Similar to another study [21], we found that physicians with a higher workload on the day of the encounter had lower billing diagnosis accuracy. We also found that claims for less complex patients (i.e., younger and less socially deprived patients) were more likely to be confirmed as syndrome-positive by the medical chart, as compared to those of more complex patients. These findings may be due to higher physician workload and greater patient complexity increasing demands on limited physician resources, taxing working memory and increasing cognitive load, thereby increasing the likelihood of physician errors, including errors in billing diagnosis. Similar to prior studies' finding that common billing diagnoses are more likely to be accurate than rare ones [31,32], we found that syndrome-positive diagnoses in physician claims were more likely to represent true-positives when the physician had billed several visits for the same syndrome recently. The observation that billing diagnosis accuracy increases with frequency of use can be explained by widely accepted theories on the effect of repetition on recall [40].

We found that billing software had a significant impact on the PPV of syndromic surveillance case definitions: billing diagnoses abstracted from the electronic medical record in an automated manner were more accurate than diagnoses input manually for billing purposes. Although this finding is based upon only a few approaches that we were able to categorize as automatic or manual, it has important implications for both clinical users and public health surveillance. Whereas public health surveillance previously required health practitioners to submit case reports manually, it is now becoming a process where public health agencies automatically extract relevant data from clinical information systems. Indeed, the US federal government has allotted $39 billion to support the adoption and 'meaningful use' of electronic health records, and software purchased using these funds must support automated submission of data to public health agencies for three public health uses, including syndromic surveillance [41]. This investment presents an opportunity to improve syndromic surveillance systems by having electronic health records capture and transmit information on highly influential predictors of case definition accuracy. To this end, a working group of surveillance experts from the US Centers for Disease Control and Prevention and the International Society for Disease Surveillance recently proposed specifications for the data captured by emergency department electronic health records and transmitted to public health [42]; however, this process has yet to take place for community-based ambulatory care settings. Our study findings are directly relevant to the discussion of what data elements should be captured and transmitted by electronic health records from primary care settings to public health under the 'meaningful use' mandate.

Our study had several strengths. It was based on a large representative sample of physicians and patients. We had access to many physician, patient, encounter, and billing characteristics, which enabled us to perform a comprehensive assessment of the impact of a variety of factors on the accuracy of syndromic surveillance case definitions. Whereas some of our findings may be specific to our study population, most of our findings are likely generalizable across North American jurisdictions due to similar physician and patient populations. A limitation of our study was that the number of visits per syndrome was too small to identify predictors of case definition accuracy specific to each syndrome individually. Whereas most of the predictors of case definition accuracy that we identified would be expected to impact all syndrome definitions in a similar manner (e.g., physician workload, patient complexity), some predictors (e.g., season) may have a greater impact on some case definitions than others. Also, it should be noted that our study identified predictors of the PPV of billing diagnoses; therefore, our findings may not be directly applicable to surveillance systems that use different data, such as chief complaints from emergency departments. However, the research methodology described in this manuscript can be used to identify predictors of accuracy of other types of surveillance data.

## Conclusions

Through a chart validation involving a large random sample of physicians, we have demonstrated that measurable elements of the medical encounter affect the accuracy of syndrome reports derived from physician claims. These elements, which include physician, patient, encounter, and billing characteristics, can be collected by public health departments through automated surveillance systems and used to focus or adjust analyses in order to reduce false-alerts. The rich clinical data streams becoming accessible to public health should enable the implementation of surveillance strategies that incorporate our findings. As the volume and detail of clinical data continue to increase, future research should explore how public health can harness their full breadth to further enhance the accuracy of case detection.

## Competing interests

The authors declare that they have no competing interests.

## Authors' contributions

All authors read and approved the final manuscript. GC collected the data, performed the data analysis, and is the primary author of the manuscript. AJ helped develop the methods and collect the data, and provided useful comments on the manuscript. ML helped develop the methods and provided useful comments on the manuscript. ND helped develop the methods, led the analysis, and provided useful comments on the manuscript. RT and DLB provided access to claims data and study subjects, helped develop the methods, oversaw the analysis, and provided useful comments on the manuscript.

## Pre-publication history

The pre-publication history for this paper can be accessed here:

http://www.biomedcentral.com/1471-2458/12/166/prepub

## Supplementary Material

Additional file 1 **Table S1**. Studies that have identified covariates associated with the accuracy of chronic disease case definitions based on diagnoses in administrative data. Click here for file

Additional file 2 **Table S2**. Patient, physician, encounter, and billing characteristics associated with accuracy of syndrome definitions based on physician claims: results from bivariate regression analyses for each syndrome individually. Click here for file
