# Supplementary material for: Patient, physician, encounter, and billing characteristics predict the accuracy of syndromic surveillance case definitions
Source: BMC Public Health. 2012 Mar 8;12:166. doi: 10.1186/1471-2458-12-166 (PMC3378465; doi:10.1186/1471-2458-12-166)
Supplement: Additional file 1 — Table S1. Studies that have identified covariates associated with the accuracy of chronic disease case definitions based on diagnoses in administrative data. [file 1471-2458-12-166-S1.DOC]

**Table 1. Studies that have identified covariates associated with the accuracy of case definitions based on diagnoses in administrative data**

| **Study** | **Disease(s) under study** | **Administrative data being validated (format)** | **Data used as comparison (format)** | **Covariate data source** | **Study population**  **and sampling** | **Statistical analysis** | **Findings** |
| --- | --- | --- | --- | --- | --- | --- | --- |
| Farzandipour *et al*., 2010 [1] | No specific disease targeted | Hospital discharge diagnoses (ICD-10 codes) | Blinded expert recoding of discharge diagnoses (ICD-10 codes) | Original coder’s experience and education were obtained from personnel files  Coding practice was assessed through direct observation | Stratified random sample of 370 hospitalizations to 4 teaching hospitals in Kashan, Iran in 2007-2008 | Outcome: errors in principal diagnosis coding  Bivariate analyses  Chi-squared tests | Errors were present in principal diagnosis coding in 84 (22.7%) medical records.  Less experienced coders made fewer errors (p <0.0001)  As compared to memory-based coding, using reference materials was associated with fewer coding errors (p<0.0001). |
| Studney *et al*., 1981 [2] | No specific disease targeted | Billing diagnoses on account cards (in words, not coded) | Medical record review | Average patients seen per day was computed from day sheets for the study period  Gross income was based on gross billing for the study period | Sample of 1,215 visits to 12 physicians from 1 clinic located in British Columbia, Canada over a 3-month period in 1976 | Outcome: % agreement between billing diagnosis and medical record diagnosis  Bivariate analyses  Pearson’s correlation coefficients | Disagreement on principal diagnosis was present in 40% of visits.  Agreement was significantly (p<0.01) and negatively associated with physician workload, as measured by both average patients seen per day (r = -0.73) and gross income (r=-0.75). |
| Peabody *et al*., 2004 [3] | Diabetes, COPD, vascular disease, depression | 1) Diagnoses in computerized administrative records (format not described)  2) Diagnoses on administrative encounter forms | Medical record review | Disease was assigned to each standardized patient  Training was categorized as 2^nd^ year resident, 3^rd^ year resident, or attending physician | 348 visits by 45 standardized patients at 3 outpatient clinics in San Francisco, CA between March-July 2002 | Outcome: % agreement between diagnoses in administrative data and medical record diagnoses  Bivariate analyses  Chi-squared tests | Administrative data contained the correct diagnosis in 57% of visits.  Agreement was significantly (p<0.05) different for different diseases and clinics.  Agreement was not significantly associated with physician training (R2, R3, or attending). |
| Kostylova *et al*., 2005 [4] | Head injury, probable head injury, orthopedic injury | Physician claims diagnoses from the *Régie de l’assurance maladie du Québec* (ICD-9 codes) | Canadian Hospitals Injury research and Prevention Program (CHIRPP) database | Patient age and sex was obtained from the CHIRPP database | 3,145 children aged 1-18 years who sought care for an injury in 2000-2001 at either of the two Montreal pediatric hospitals emergency departments | Outcome: agreement between physician claims diagnosis and CHIRPP data  Bivariate analyses | Good agreement (Kappa, 0.66)  Mean age of children whose claim diagnosis disagreed with CHIRPP diagnosis (6.6 years) was significantly lower than that of children whose claim diagnosis agreed with the CHIRPP diagnosis (8.5 years) (p<0.001). |

**Table 1 (continued). Studies that have identified covariates associated with the accuracy of case definitions based on diagnoses in administrative data**

| **Study** | **Disease(s) under study** | **Administrative data being validated (format)** | **Data used as comparison (format)** | **Covariate data source** | **Study population**  **and sampling** | **Statistical analysis** | **Findings** |
| --- | --- | --- | --- | --- | --- | --- | --- |
| Andrade *et al*., 2002 [5] | Peptic ulcer, upper gastro-intestinal bleeding | Hospital discharge diagnoses (ICD-9-CM codes) | Diagnosis confirmed by surgery, endoscopy, x-ray or autopsy documented in the hospital chart | Patient age and gender were obtained from the health maintenance organization database | 8 large US health maintenance organizations  1,152 hospitalizations with a hospital discharge diagnosis of peptic ulcer or upper gastrointestinal bleeding in 1994-1997  The hospital chart was located for 884 (76.7%) hospitalizations | Outcome: PPV of hospital discharge diagnoses  Bivariate analyses  Chi-squared test | The discharge diagnosis was confirmed for 207 (23.4%) hospitalizations.  The proportion of confirmed discharge diagnoses was higher among patients aged less than 60 years (32%) as compared to those 80 years and older (19%) (p = 0.01).  Site-specific diagnostic codes had higher positive predictive values than nonspecific codes. |
| Losina et al., 2003 [6] | Rheumatoid arthritis, avascular necrosis, osteoathritis | Hospital and surgeons’ Medicare claim diagnoses (ICD-9-CM codes) | Medical record review | Hospitals were categorized as low- and high-volume based on a cut-off of 25 total hip replacements per year (median of the sample) | A stratified random sample of 922 Medicare beneficiaries who received total hip replacement in 1995 in Ohio, Pennsylvania, and Colorado | Outcomes: sensitivity and PPV of the Medicare claim diagnoses  Bivariate analyses  Chi-squared test (p<0.05) | Sensitivity ranged from  0.54 (95% CI, 0.42-0.66) for avascular necrosis to 0.96 (95% CI, 0.95-0.98) for osteoathritis.  PPV did not differ by disease (range, 0.86-0.89).  Sensitivity was higher for rheumatoid arthritis and lower for avascular necrosis in low-volume hospitals as compared to high-volume ones.  No relationship between PPV and hospital volume. |
| Jollis *et al*., 1993 [7] | 12 prognostic factors in patients with ischemic heart disease | Medicare claims diagnoses (ICD-9-CM codes) | Hospital clinical information system | Patient age and sex were obtained from the hospital clinical information system | All 12,937 Medicare patients who underwent inpatient cardiac catheterization at Duke Medical Center in 1985-1990 | Outcome: Sensitivity of Medicare claims diagnoses  Bivariate analyses  Chi-squared test | The sensitivity of Medicare claims diagnoses ranged from 0.83 for diabetes to 0.14 for unstable angina and tobacco use.  Sensitivity of Medicare claims diagnoses was significantly higher for patients aged less than 65 years as compared to patients aged 65 years and older. |
| McClish *et al*., 1997 [8] | Breast,  colorectal, lung, and prostate cancers | Medicare claims diagnoses (ICD-9-CM codes) | Virginia Cancer Registry | Patient age, gender, and comorbidity were obtained from the Medicare database  Income and education were obtained from the 1990 Census  Cancer stage was obtained from the Virginia Cancer Registry | All Virginia residents aged 65 years and older with a breast (N=3,690), colorectal (N=4,690), lung (N=5,781), or prostate (N=4,495) cancer diagnosis in the Virginia Cancer Registry in 1986-1989 | Outcome: case not identified by Medicare claims (yes/no)  Multivariate logistic regression analyses stratified by type of cancer | Medicare claims diagnoses identified between 73% (prostate cancer) and 83% (breast and lung cancers) of cases in the registry.  Patient characteristics associated with Medicare claims data missing a case included age 65-75 years (vs. 75+ years), male gender, urban area of residence, higher education and income, in situ disease, and lack of comorbidity. |

**Table 1 (continued). Studies that have identified covariates associated with the accuracy of case definitions based on diagnoses in administrative data**

| **Study** | **Disease(s) under study** | **Administrative data being validated (format)** | **Data used as comparison (format)** | **Covariate data source** | **Study population**  **and sampling** | **Statistical analysis** | **Findings** |
| --- | --- | --- | --- | --- | --- | --- | --- |
| Ostbye *et al*., 2008 [9] | Dementia | Medicare claims diagnoses (ICD-9-CM codes) | Self- or proxy-reported cognitive status from the Asset and Health Dynamics among the Oldest Old (AHEAD) national survey | Patient age, gender, and education (data source not described) | 7,974 cases of dementia were identified from the AHEAD study  Of those, 80% consented to share their Medicare claims data with the researchers | Outcome: agreement (yes/no)  Multivariate logistic regression analyses with | Agreement between Medicare claims and survey data was poor (Cohen’s Kappa, 0.23; 95% CI, 0.17-0.30).  Agreement between Medicare claims and survey data was more likely among older (OR_per year of age_, 0.94; 95% CI, 0.93-0.96) and more educated respondents (OR_per year od education_ 1.05; 95% CI, 1.02-1.07). |
| Muhajarine *et al*., 1997 [10] | Hypertension | Physician claims data from the Manitoba Health Insurance Plan (ICD-9-CM codes) | Manitoba Heart Health Survey:  1) patient self-report  2) clinical measurement of blood pressure | Patient sex, age, education, income, employment, and marital status, smoking, obesity, cholesterol levels, physical activity, diabetes, and cardiac medications were obtained from the survey | Stratified random sample of 2,792 non-institutionalized adults aged 18-74 years residing in Manitoba  2,339 (84%) completed the clinical visit  2,275 (97.3%) had a physician claim in the 2 years before the survey | Outcome: Disagreement (yes/no)  Multivariate logistic regression analyses | Disagreement between claims and clinical measurement was more likely among homemakers (OR, 1.78; 95% CI, 1.11-2.84), 1.62-2.79), obese patients (OR, 2.13; 95% CI, 1.62-2.79), older patients (OR _55+ vs. 18-34 y.o._, 2.92; 95% CI, 1.84-4.61), patients with hypercholesterolemia (OR, 1.35; 95% CI 1.02-1.79), and patients on medication for heart disease (OR, 1.66; 95% CI 1.13-2.43). |
| MacIntyre *et al*., 1997 [11] | Australian national diagnosis-related groups (ANDRG) | Victorian In-Patient Minimum Database (ICD-9-CM codes) | Medical record audit | Hospital location (rural or urban), admission type (emergency or other), length of stay, death, ANDRG frequency, and type of ANDRG (medical or other) were obtained from the Victorian In-Patient Minimum Database | 7,013 cases from 63 (54%) Victorian hospitals in the year 1993-1994 were randomly selected and audited | Outcome: disagreement on principal diagnosis  Multivariate logistic regression analyses | Disagreement was present in 1,565 (22%) cases.  Disagreement was more likely among hospitalizations longer than 5 days (OR, 1.95; 95% CI, 1.69-2.25), involving emergency admission (OR, 1.14; (% CI, 1.02-1.28), and resulting in death (OR, 1.90; 95% CI 1.19-3.00).  Likelihood of discrepancy increased with increasing rarity of ANDRG. |
| Taylor *et al*., 2002 [12] | Alzheimer’s disease | ICD-9 codes in Medicare claims (ICD-9 codes) | Clinical diagnosis of Alzheimer’s disease in the Consortium to Establish a Registry for Alzheimer’s Disease (CERAD) | Patient age, education, marital status, and Alzheimer’s disease severity, were obtained from CERAD  Number of visits was ascertained from Medicare claims | 417 patients with a clinical diagnosis of Alzheimer’s disease in CERAD | Outcome: Alzheimer’s disease diagnosis in Medicare claims (among patient so diagnosed in CERAD)  Multivariate Cox proportional hazards analyses | A diagnosis of Alzheimer’s in Medicare claims was more likely among males (HR, 1.27; p<0.05), younger patients (HR_per year_, 0.97; p<0.05), patients with less severe disease (HR_stage_, 0.65; p<0.01).  The likelihood of a diagnosis of Alzheimer’s in Medicare claims increased with the number of visits (HR_per visit_, 1.09; p<0.001). |

**Table 1 (continued). Studies that have identified covariates associated with the accuracy of case definitions based on diagnoses in administrative data**

| **Study** | **Disease(s) under study** | **Administrative data being validated (format)** | **Data used as comparison (format)** | **Covariate data source** | **Study population**  **and sampling** | **Statistical analysis** | **Findings** |
| --- | --- | --- | --- | --- | --- | --- | --- |
| Singh *et al*., 2009 [13] | Arthritis | Diagnosis in the Veteran’s Affairs administrative data (ICD-9 -CM codes) | Patient self-reported physician diagnosis from the Prior Veterans’ Quality of Life (Vet-QoL) survey | Patient sex, education, comorbidity, activity limitation, tobacco use, and health status (SF-36) were obtained from the survey database | Of 70,334 veterans contacted for the survey, 34,440 (49.0%) answered the question on arthritis | Outcome: Discordance between administrative and survey data  Multivariate logistic regression analyses | Poor agreement (Kappa, 0.25)  Discordance more likely among infrequent healthcare users (OR_lowest vs. highest tertile_, 1.11; 95% CI, 1.03-1.19), with no prior hospitalization (OR_1+ vs. 0_, 0.88; 95% CI, 0.80-0.96), older veterans (OR_65+ vs. <50 y.o._, 1.38; 95% CI, 1.26-1.52), with more comorbidities (OR_3+ vs. 0_, 1.54; 95% CI, 1.40-1.69), and worse physical health (OR_highest vs. lowest PCS score tertile_, 0.83; 95% CI, 0.76-0.91). |
| Lix *et al*., 2008 [14] | Osteoporosis | Physician claims diagnoses from the Manitoba Health Insurance Plan (ICD-9-CM codes) | BMD test results in the Manitoba Bone Density Program database | Patient age, region of residence, income quintile, comorbidity, osteoporosis prescriptions were obtained from administrative databases | 5,527 patients with at least one BMD test in the Manitoba Bone Density Program database during the fiscal year 2000-2001 | Outcome: osteoporosis based on BMD test result  Multivariate logistic regression analyses | Likelihood of osteoporosis based on BMD test increased with an ICD-9 diagnostic code for osteoporosis in physician claims (OR, 2.74; p <0.0001), a prescription for an osteoporosis-specific medication (OR, 5.13; p<0.0001), and older age (OR_per year of age_: 1.06; p<0.0001). |
| Gabriel *et al*., 1996 [15] | Osteoarthritis | Diagnoses from the Olmsted County, Minnesota computerized database (ICD-9 code) | Medical record review | Patient age and sex were obtained from the computerized database | Random sample of 400 patients with a diagnosis of osteoarthritis in the computerized database in1975-1987  The medical record was available for 387 (96.8%) patients | Outcome: positive predictive value of the computerized database  Recursive partitioning analysis | Positive predictive value of the computerized database: 60% (232/387).  Older patients were more likely to be true-positives. |
| Love *et al*., 2010 [16] | Psoriatic arthritis | Physician billing diagnostic codes (ICD-9) in the Research Patient Data Registry (RDPR) | Rheumatologist-diagnosed psoriatic arthritis ascertained by a review of the RDPR | Coded data from the RDPR  Information extracted from the full text visit notes from the RDPR  Laboratory values from the RDPR | 2,318 patients with a physician billing diagnostic code for psoriatic arthritis in 1995-2007 were identified from the RDPR of the Brigham and Women’s Hospital and its outpatient clinics (Boston, MA)  A random sample of 550 (23.7%) patient records were reviewed | Outcome: rheumatologist-diagnosed psoriatic arthritis  Random forest analysis (an extension of recursive partitioning) | The sensitivity of coded data (e.g., number of psoriatic arthritis diagnostic codes) for identifying rheumatologist-diagnosed psoriatic arthritis was improved by the addition of variables extracted from the medical record using natural language processing (e.g., the number of rheumatology and clinic notes mentioning psoriatic arthritis) and laboratory values (e.g., highest erythrocyte sedimentation rate). |

**Table 1 (continued). Studies that have identified covariates associated with the accuracy of case definitions based on diagnoses in administrative data**

| **Study** | **Disease(s) under study** | **Administrative data being validated (format)** | **Data used as comparison (format)** | **Covariate data source** | **Study population**  **and sampling** | **Statistical analysis** | **Findings** |
| --- | --- | --- | --- | --- | --- | --- | --- |
| Szumski *et al*., 2009 [17] | Parkinson’s disease | Diagnoses from Veteran’s Affairs administrative data (ICD-9-CM codes) | Medical record review | Patient age, sex, number of diagnostic codes for Parkinson’s disease, number of visits to neurology clinic, and number of visits to movement disorder clinic were obtained from the Veteran’s Affairs database | 577 patients with a diagnosis of Parkinson’s disease in the Veteran’s Affairs administrative database between October 1, 2001 and September 30, 2002 | Outcome: sensitivity, specificity, PPV and NPV of administrative data as compared to the chart  Two-sample test of proportion (α=0.05) used to compare outcome estimates across algorithms | Medical record review determined 436 (75.6%) patients to have Parkinson’s disease.  PPV of administrative data was improved by giving greater weight to diagnostic codes from specialists over non-specialists, and by taking into account prescriptions for Parkinson’s disease-related medications. |
| Van Walraven *et al*., 2010 [18] | Kidney disease | Diagnoses in the Ottawa Hospital Data Warehouse (ICD-10) | Serum creatinine measurements from the from the Ottawa Hospital Data Warehouse | Patient age, sex, and comorbidity were obtained from the Ottawa Hospital Data Warehouse  Hospitalization-level characteristics: admission urgency, admitting service, ICU stay, surgical procedures, hospital survival, and length of stay were obtained from the Ottawa Hospital Data Warehouse | A random sample of 100,000 adult admissions to the Ottawa Hospital in 2002-2008 was selected from the Ottawa Hospital Data Warehouse | Outcome: kidney disease based on serum creatinine measurements  Multivariate logistic regression | Kidney disease was confirmed by serum creatinine in 20,713 (20.7%) patients.  The most powerful predictor of kidney disease based on serum creatinine was a diagnostic code for kidney disease (RR: 34.4).  Other variables were associated with kidney disease based on serum creatinine, including patient characteristics (e.g., age, gender, comorbidities) and hospitalization characteristics (e.g., length of stay, ICU stay, dialysis). |

REFERENCES

1. Farzandipour M, Sheikhtaheri A, Sadoughi F: **Effective factors on accuracy of principal diagnosis coding based on International Classification of Diseases, the 10th revision (ICD-10).** *Int J Inf Manage* 2010, **30:** 78-84.

2. Studney DR, Hakstian AR: **A Comparison of Medical Record with Billing Diagnostic Information Associated with Ambulatory Medical-Care.** *Am J Public Health* 1981, **71:** 145-149.

3. Peabody JW, Luck J, Jain S, Bertenthal D, Glassman P: **Assessing the accuracy of administrative data in health information systems.** *Medical Care* 2004, **42:** 1066-1072.

4. Kostylova A, Swaine B, Feldman D: **Concordance between childhood injury diagnoses from two sources: an injury surveillance system and a physician billing claims database.** *Inj Prev* 2005, **11:** 186-190.

5. Andrade SE, Gurwitz JH, Chan KA, Donahue JG, Beck A, Boles M *et al*.: **Validation of diagnoses of peptic ulcers and bleeding from administrative databases: A multi-health maintenance organization study.** *J Clin Epidemiol* 2002, **55:** 310-313.

6. Losina E, Barrett J, Baron JA, Katz JN: **Accuracy of Medicare claims data for rheumatologic diagnoses in total hip replacement recipients.** *J Clin Epidemiol* 2003, **56:** 515-519.

7. Jollis JG, Ancukiewicz M, Delong ER, Pryor DB, Muhlbaier LH, Mark DB: **Discordance of Databases Designed for Claims Payment Versus Clinical Information-Systems - Implications for Outcomes Research.** *Annals of Internal Medicine* 1993, **119:** 844-850.

8. McClish DK, Penberthy L, Whittemore M, Newschaffer C, Woolard D, Desch CE *et al*.: **Ability of Medicare claims data and cancer registries to identify cancer cases and treatment.** *Am J Epidemiol* 1997, **145:** 227-233.

9. Ostbye T, Taylor DH, Clipp EC, Van Scoyoc L, Plassman BL: **Identification of dementia: Agreement among national survey data, medicare claims, and death certificates.** *Health Services Research* 2008, **43:** 313-326.

10. Muhajarine N, Mustard C, Roos LL, Young TK, Gelskey DE: **Comparison of survey and physician claims data for detecting hypertension.** *J Clin Epidemiol* 1997, **50:** 711-718.

11. MacIntyre CR, Ackland MJ, Chandraraj EJ, Pilla JE: **Accuracy of ICD-9-CM codes in hospital morbidity data, Victoria: implications for public health research.** *Aust N Z J Public Health* 1997, **21:** 477-482.

12. Taylor DH, Fillenbaum GG, Ezell ME: **The accuracy of medicare claims data in identifying Alzheimer's disease.** *J Clin Epidemiol* 2002, **55:** 929-937.

13. Singh JA: **Discordance Between Self-report of Physician Diagnosis and Administrative Database Diagnosis of Arthritis and Its Predictors.** *J Rheumatol* 2009, **36:** 2000-2008.

14. Lix LM, Yogendran MS, Leslie WD, Shaw SY, Baumgartner R, Bowman C *et al*.: **Using multiple data features improved the validity of osteoporosis case ascertainment from administrative databases.** *J Clin Epidemiol* 2008, **61:** 1250-1260.

15. Gabriel SE, Crowson CS, OFallon WM: **A mathematical model that improves the validity of osteoarthritis diagnoses obtained from a computerized diagnostic database.** *J Clin Epidemiol* 1996, **49:** 1025-1029.

16. Love TJ, Cai T, Karlson EW: **Validation of Psoriatic Arthritis Diagnoses in Electronic Medical Records Using Natural Language Processing.** *Semin Arthritis Rheum* 2010, **40:** 413-420.

17. Szumski NR, Cheng EM: **Optimizing Algorithms to Identify Parkinson's Disease Cases Within an Administrative Database.** *Mov Disord* 2009, **24:** 51-56.

18. van Walraven C, Austin PC, Manuel D, Knoll G, Jennings A, Forster AJ: **The usefulness of administrative databases for identifying disease cohorts is increased with a multivariate model.** *J Clin Epidemiol* 2010, **63:** 1332-1341.
