# Supplementary material for: Patient, physician, encounter, and billing characteristics predict the accuracy of syndromic surveillance case definitions
Source: BMC Public Health. 2012 Mar 8;12:166. doi: 10.1186/1471-2458-12-166 (PMC3378465; doi:10.1186/1471-2458-12-166)
Supplement: Additional file 2 — Table S2. Patient, physician, encounter, and billing characteristics associated with accuracy of syndrome definitions based on physician claims: results from bivariate regression analyses for each syndrome individually. [file 1471-2458-12-166-S2.DOC]

**Table 1. Physician characteristics associated with accuracy of syndrome definitions based on physician claims: results from *bivariate* regression analyses for each syndrome individually** (OR>1.00 means the physician characteristic increased the PPV of the syndrome definition, OR<1.00 means the physician characteristic reduced the PPV)

|  | **Fever syndrome**  **(N=601 visits)** | | | | **Gastrointestinal syndrome**  **(N=855 visits)** | | | | **Neurological syndrome**  **(N=971 visits)** | | | | **Rash syndrome**  **(N=897 visits)** | | | | **Respiratory syndrome**  **(N=1,049 visits)** | | | | **Influenza-like illness**  **(N=653 visits)** | | | |
| --- | --- | --- | --- | --- | --- | --- | --- | --- | --- | --- | --- | --- | --- | --- | --- | --- | --- | --- | --- | --- | --- | --- | --- | --- |
| **Physician characteristics** | **No. visits** | **OR** | **95% CI** | **P Value** | **No. visits** | **OR** | **95% CI** | **P value** | **No. visits** | **OR** | **95% CI** | **P Value** | **No. visits** | **OR** | **95% CI** | **P value** | **No. visits** | **OR** | **95% CI** | **P Value** | **No. visits** | **OR** | **95% CI** | **P value** |
| Gender: |  |  |  |  |  |  |  |  |  |  |  |  |  |  |  |  |  |  |  |  |  |  |  |  |
| Female | 249 | Ref. | Ref. | Ref. | 340 | Ref. | Ref. | Ref. | 371 | Ref. | Ref. | Ref. | 338 | Ref. | Ref. | Ref. | 401 | Ref. | Ref. | Ref. | 255 | Ref. | Ref. | Ref. |
| Male | 352 | 0.82 | (0.60, 1.14) | 0.25 | 515 | 0.83 | (0.62, 1.11) | 0.21 | 600 | 1.09 | (0.83, 1.41) | 0.54 | 559 | 1.37 | (1.03, 1.84) | 0.03 | 648 | 0.72 | (0.54, 0.98) | 0.04 | 398 | 0.93 | (0.64, 1.33) | 0.67 |
| Preferred language: |  |  |  |  |  |  |  |  |  |  |  |  |  |  |  |  |  |  |  |  |  |  |  |  |
| French | 548 | Ref. | Ref. | Ref. | 784 | Ref. | Ref. | Ref. | 901 | Ref. | Ref. | Ref. | 830 | Ref. | Ref. | Ref. | 973 | Ref. | Ref. | Ref. | 595 | Ref. | Ref. | Ref. |
| English | 53 | 0.59 | (0.33, 1.05) | 0.07 | 71 | 0.79 | (0.48, 1.30) | 0.35 | 70 | 1.13 | (0.68, 1.86) | 0.64 | 67 | 1.22 | (0.70, 2.14) | 0.48 | 76 | 0.85 | (0.50, 1.44) | 0.54 | 58 | 0.85 | (0.47, 1.56) | 0.61 |
| Specialty: |  |  |  |  |  |  |  |  |  |  |  |  |  |  |  |  |  |  |  |  |  |  |  |  |
| General practice | 539 | Ref. | Ref. | Ref. | 776 | Ref. | Ref. | Ref. | 899 | Ref. | Ref. | Ref. | 828 | Ref. | Ref. | Ref. | 961 | Ref. | Ref. | Ref. | 575 | Ref. | Ref. | Ref. |
| Internal medicine | 8 | 0.30 | (0.06, 1.50) | 0.14 | 12 | 0.18 | (0.05, 0.68) | 0.01 | 11 | 0.54 | (0.16, 1.79) | 0.31 | 8 | 0.72 | (0.17, 3.02) | 0.65 | 11 | 3.16 | (0.40, 24.83) | 0.27 | 9 | 0.67 | (0.17, 2.73) | 0.58 |
| Pediatrics | 51 | 1.51 | (0.84, 2.73) | 0.17 | 54 | 1.57 | (0.84, 2.93) | 0.16 | 56 | 1.00 | (0.59, 1.75) | 0.99 | 55 | 0.82 | (0.46, 1.45) | 0.49 | 67 | 2.04 | (1.00, 4.18) | 0.05 | 65 | 1.49 | (0.77, 2.87) | 0.23 |
| General surgery | 3 | >999 | infinity | 0.98 | 13 | 0.34 | (0.11, 1.06) | 0.06 | 5 | 0.16 | (0.02, 1.46) | 0.10 | 6 | 0.22 | (0.04, 1.18) | 0.08 | 10 | 0.32 | (0.09, 1.10) | 0.07 | 4 | 0.34 | (0.05, 2.42) | 0.28 |
| Years since licensure (per 5 years) | 601 | 0.92 | (0.84, 1.00) | 0.05 | 855 | 0.91 | (0.84, 0.98) | 0.01 | 971 | 0.99 | (0.92, 1.06) | 0.76 | 897 | 0.97 | (0.89, 1.04) | 0.35 | 1,049 | 0.93 | (0.86, 1.01) | 0.07 | 653 | 0.93 | (0.84, 1.02) | 0.12 |

**Table 2. Patient characteristics associated with accuracy of syndrome definitions based on physician claims: results from *bivariate* regression analyses for each syndrome individually** (OR>1.00 means the patient characteristic increased the PPV of the syndrome definition, OR<1.00 means the patient characteristic reduced the PPV)

|  | **Fever syndrome**  **(N=601 visits)** | | | | **Gastrointestinal syndrome**  **(N=855 visits)** | | | | **Neurological syndrome**  **(N=971 visits)** | | | | **Rash syndrome**  **(N=897 visits)** | | | | **Respiratory syndrome**  **(N=1,049 visits)** | | | | **Influenza-like illness**  **(N=653 visits)** | | | |
| --- | --- | --- | --- | --- | --- | --- | --- | --- | --- | --- | --- | --- | --- | --- | --- | --- | --- | --- | --- | --- | --- | --- | --- | --- |
| **Patient characteristics** | **No. visits** | **OR** | **95% CI** | **P Value** | **No. visits** | **OR** | **95% CI** | **P value** | **No. visits** | **OR** | **95% CI** | **P value** | **No. visits** | **OR** | **95% CI** | **P value** | **No. visits** | **OR** | **95% CI** | **P value** | **No. visits** | **OR** | **95% CI** | **P value** |
| Sex: |  |  |  |  |  |  |  |  |  |  |  |  |  |  |  |  |  |  |  |  |  |  |  |  |
| Female | 366 | Ref. | Ref. | Ref. | 529 | Ref. | Ref. | Ref. | 642 | Ref. | Ref. | Ref. | 534 | Ref. | Ref. | Ref. | 585 | Ref. | Ref. | Ref. | 371 | Ref. | Ref. | Ref. |
| Male | 235 | 1.10 | (0.79, 1.53) | 0.56 | 326 | 0.95 | (0.71, 1.27) | 0.73 | 329 | 0.72 | (0.55, 0.94) | 0.02 | 363 | 0.85 | (0.64, 1.13) | 0.26 | 464 | 1.00 | (0.75, 1.33) | 0.98 | 282 | 1.45 | (1.01, 2.10) | 0.05 |
| Material deprivation index:^1^ | 601 | 1.18 | (1.05, 1.34) | <0.01 | 855 | 1.06 | (0.96, 1.17) | 0.28 | 971 | 1.03 | (0.94, 1.13) | 0.55 | 897 | 1.08 | (0.97, 1.20) | 0.14 | 1,049 | 0.96 | (0.87, 1.06) | 0.43 | 653 | 1.18 | (1.03, 1.34) | 0.01 |
| 1^st^ quintile (least deprived) | 107 | Ref. | Ref. | Ref. | 153 | Ref. | Ref. | Ref. | 177 | Ref. | Ref. | Ref. | 174 | Ref. | Ref. | Ref. | 204 | Ref. | Ref. | Ref. | 140 | Ref. | Ref. | Ref. |
| 2^nd^ quintile | 121 | 1.39 | (0.83, 2.35) | 0.21 | 152 | 1.41 | (0.89, 2.24) | 0.14 | 183 | 1.19 | (0.78, 1.81) | 0.42 | 196 | 1.29 | (0.83, 1.96) | 0.25 | 213 | 0.88 | (0.56, 1.38) | 0.57 | 126 | 1.24 | (0.73, 2.11) | 0.43 |
| 3^rd^ quintile | 129 | 1.53 | (0.91, 2.56) | 0.11 | 173 | 1.63 | (1.04, 2.56) | 0.03 | 196 | 1.52 | (1.00, 2.31) | 0.05 | 176 | 1.47 | (0.94, 2.31) | 0.09 | 180 | 1.17 | (0.71, 1.93) | 0.53 | 115 | 1.42 | (0.82, 2.48) | 0.21 |
| 4^th^ quintile | 121 | 1.54 | (0.91, 2.60) | 0.11 | 171 | 1.64 | (1.05, 2.58) | 0.03 | 181 | 1.19 | (0.78, 1.82) | 0.41 | 170 | 1.36 | (0.87, 2.14) | 0.18 | 208 | 0.89 | (0.57, 1.42) | 0.63 | 130 | 1.67 | (0.96, 2.89) | 0.07 |
| 5^th^ quintile (most deprived) | 92 | 2.26 | (1.28, 4.00) | 0.01 | 168 | 1.24 | (0.79, 1.93) | 0.35 | 203 | 1.16 | (0.77, 1.75) | 0.47 | 147 | 1.42 | (0.88, 2.28) | 0.15 | 196 | 0.81 | (0.51, 1.27) | 0.35 | 109 | 1.92 | (1.06, 3.48) | 0.03 |
| Missing | 31 | 2.78 | (1.20, 6.48) | 0.02 | 38 | 2.51 | (1.11, 5.66) | 0.03 | 31 | 0.96 | (0.44, 2.06) | 0.91 | 34 | 1.19 | (0.54, 2.60) | 0.67 | 48 | 0.76 | (0.37, 1.56) | 0.46 | 33 | 1.70 | (0.69, 4.22) | 0.25 |
| Social deprivation index:^1^ | 601 | 0.98 | (0.87, 1.11) | 0.79 | 855 | 0.94 | (0.85, 1.04) | 0.21 | 971 | 0.97 | (0.88, 1.06) | 0.52 | 897 | 0.95 | (0.85, 1.05) | 0.27 | 1,049 | 0.92 | (0.83, 1.02) | 0.13 | 653 | 0.94 | (0.83, 1.07) | 0.34 |
| 1^st^ quintile (least deprived) | 106 | Ref. | Ref. | Ref. | 162 | Ref. | Ref. | Ref. | 192 | Ref. | Ref. | Ref. | 197 | Ref. | Ref. | Ref. | 215 | Ref. | Ref. | Ref. | 130 | Ref. | Ref. | Ref. |
| 2^nd^ quintile | 131 | 0.87 | (0.52, 1.46) | 0.60 | 163 | 1.01 | (0.64, 1.60) | 0.97 | 197 | 1.11 | (0.74, 1.67) | 0.61 | 170 | 0.61 | (0.39, 0.95) | 0.03 | 184 | 1.22 | (0.75, 1.99) | 0.42 | 134 | 1.09 | (0.62, 1.91) | 0.78 |
| 3^rd^ quintile | 117 | 0.97 | (0.57, 1.64) | 0.91 | 162 | 1.12 | (0.70, 1.77) | 0.64 | 202 | 1.16 | (0.77, 1.74) | 0.48 | 167 | 0.73 | (0.46, 1.16) | 0.19 | 184 | 0.89 | (0.56, 1.41) | 0.62 | 102 | 1.01 | (0.55, 1.84) | 0.99 |
| 4^th^ quintile | 109 | 1.27 | (0.74, 2.18) | 0.39 | 163 | 0.70 | (0.45, 1.10) | 0.12 | 169 | 1.12 | (0.74, 1.72) | 0.59 | 164 | 0.61 | (0.38, 0.96) | 0.03 | 214 | 0.99 | (0.63, 1.57) | 0.98 | 133 | 1.17 | (0.66, 2.08) | 0.59 |
| 5^th^ quintile (most deprived) | 107 | 0.75 | (0.44, 1.29) | 0.30 | 167 | 0.87 | (0.56, 1.37) | 0.55 | 180 | 0.86 | (0.57, 1.29) | 0.46 | 165 | 0.76 | (0.48, 1.22) | 0.25 | 204 | 0.74 | (0.48, 1.16) | 0.19 | 121 | 0.71 | (0.41, 1.24) | 0.23 |
| Missing | 31 | 1.81 | (0.78, 4.20) | 0.17 | 38 | 1.70 | (0.75, 3.84) | 0.20 | 31 | 0.83 | (0.39, 1.78) | 0.63 | 34 | 0.67 | (0.31, 1.48) | 0.33 | 48 | 0.77 | (0.38, 1.58) | 0.48 | 33 | 1.21 | (0.48, 3.06) | 0.68 |
| Age (per 5 years):^2^ | 601 | 0.99 | (0.98, 1.00) | <0.01 | 855 | 0.99 | (0.98, 0.99) | <0.01 | 971 | 0.99 | (0.99, 1.00) | 0.01 | 897 | 1.00 | (0.99, 1.01) | 0.93 | 1,049 | 0.98 | (0.97, 0.98) | <0.01 | 653 | 1.00 | (0.99, 1.00) | 0.14 |
| Health care services utilization (no. ambulatory care visits in the previous year)^3^ | 601 | 0.99 | (0.97, 1.01) | 0.24 | 855 | 0.98 | (0.97, 1.00) | 0.01 | 971 | 1.00 | (0.99, 1.01) | 0.45 | 897 | 1.00 | (0.98, 1.01) | 0.50 | 1,049 | 0.97 | (0.96, 0.98) | <0.01 | 653 | 0.98 | (0.96, 1.00) | 0.02 |
| Charlson comorbidity index (per 1-point increase in score)^3^ | 601 | 0.88 | (0.75, 1.04) | 0.13 | 855 | 0.86 | (0.77, 0.97) | <0.01 | 971 | 0.96 | (0.83, 1.09) | 0.50 | 897 | 0.96 | (0.83, 1.11) | 0.59 | 1,049 | 0.84 | (0.74, 0.95) | <0.01 | 653 | 0.96 | (0.83, 1.12) | 0.63 |

^1^ The material and social deprivation indices were calculated using Statistics Canada’s 2006 census data. These indices were developed by the Quebec National Public Health Institute. The material deprivation index summarizes information on the proportion of persons who have no high school diploma, the proportion of persons employed, and the average income in the patient’s 6-digit postal code area of residence. The social deprivation index summarizes information on the proportion of single-parent families, the proportion of persons living alone, and the proportion of persons separated, divorced, or widowed in the patient’s 6-digit postal code area of residence.

^2^ On October 1^st^ of the study year when the visit took place. The study spanned 2 years: October 1, 2005 to September 30, 2006, and October 1, 2006 to September 30, 2007.

^3^ Based on all medical services claims billed by all Quebec physicians (not only the 3,600 study physicians) in the year prior to the date of the syndrome-positive visit.

**Table 3. Encounter characteristics associated with accuracy of syndrome definitions based on physician claims: results from *bivariate* regression analyses for each syndrome individually** (OR>1.00 means the encounter characteristic increased the PPV of the syndrome definition, OR<1.00 means the encounter characteristic reduced the PPV)

|  | **Fever syndrome**  **(N=601 visits)** | | | | **Gastrointestinal syndrome**  **(N=855 visits)** | | | | **Neurological syndrome**  **(N=971 visits)** | | | | **Rash syndrome**  **(N=897 visits)** | | | | **Respiratory syndrome**  **(N=1,049 visits)** | | | | **Influenza-like illness**  **(N=653 visits)** | | | |
| --- | --- | --- | --- | --- | --- | --- | --- | --- | --- | --- | --- | --- | --- | --- | --- | --- | --- | --- | --- | --- | --- | --- | --- | --- |
| **Encounter characteristics** | **No. visits** | **OR** | **95% CI** | **P Value** | **No. visits** | **OR** | **95% CI** | **P value** | **No. visits** | **OR** | **95% CI** | **P value** | **No. visits** | **OR** | **95% CI** | **P value** | **No. visits** | **OR** | **95% CI** | **P value** | **No. visits** | **OR** | **95% CI** | **P value** |
| Type of clinic: |  |  |  |  |  |  |  |  |  |  |  |  |  |  |  |  |  |  |  |  |  |  |  |  |
| Private clinic | 587 | Ref. | Ref. | Ref. | 831 | Ref. | Ref. | Ref. | 952 | Ref. | Ref. | Ref. | 880 | Ref. | Ref. | Ref. | 1,026 | Ref. | Ref. | Ref. | 642 | Ref. | Ref. | Ref. |
| Community health center | 3 | 1.70 | (0.15, 18.89) | 0.66 | 2 | 0.27 | (0.03, 3.02) | 0.29 | 4 | 0.65 | (0.09, 4.61) | 0.66 | 4 | 0.15 | (0.02, 1.40) | 0.10 | 4 | >999 | infinity | 0.98 | 3 | >999 | infinity | 0.99 |
| Hospital-based  ambulatory clinic | 11 | 0.19 | (0.04, 0.88) | 0.03 | 31 | 0.50 | (0.21, 1.18) | 0.11 | 15 | 0.32 | (0.11, 0.95) | 0.04 | 13 | 0.70 | (0.23, 2.15) | 0.53 | 19 | 0.66 | (0.24, 1.76) | 0.41 | 8 | 0.33 | (0.08, 1.32) | 0.12 |
| Geographic location of clinic: |  |  |  |  |  |  |  |  |  |  |  |  |  |  |  |  |  |  |  |  |  |  |  |  |
| Urban | 514 | Ref. | Ref. | Ref. | 720 | Ref. | Ref. | Ref. | 818 | Ref. | Ref. | Ref. | 751 | Ref. | Ref. | Ref. | 880 | Ref. | Ref. | Ref. | 560 | Ref. | Ref. | Ref. |
| Rural | 87 | 1.09 | (0.69, 1.72) | 0.72 | 135 | 1.34 | (0.90, 1.99) | 0.15 | 153 | 1.39 | (0.96, 2.00) | 0.08 | 146 | 1.26 | (0.85, 1.87) | 0.26 | 169 | 0.95 | (0.65, 1.39) | 0.79 | 93 | 0.69 | (0.43, 1.11) | 0.13 |
| Physician familiarity with the patient (patient treated by the study physician in the previous year): |  |  |  |  |  |  |  |  |  |  |  |  |  |  |  |  |  |  |  |  |  |  |  |  |
| No | 255 | Ref. | Ref. | Ref. | 307 | Ref. | Ref. | Ref. | 345 | Ref. | Ref. | Ref. | 371 | Ref. | Ref. | Ref. | 411 | Ref. | Ref. | Ref. | 295 | Ref. | Ref. | Ref. |
| Yes | 346 | 0.95 | (0.69, 1.32) | 0.77 | 548 | 0.80 | (0.60, 1.08) | 0.14 | 626 | 0.94 | (0.72, 1.23) | 0.67 | 526 | 0.83 | (0.62, 1.11) | 0.20 | 638 | 0.45 | (0.33, 0.62) | <0.01 | 358 | 0.81 | (0.57, 1.17) | 0.26 |
| Season: |  |  |  |  |  |  |  |  |  |  |  |  |  |  |  |  |  |  |  |  |  |  |  |  |
| Winter (12/22-03/20) | 153 | Ref. | Ref. | Ref. | 211 | Ref. | Ref. | Ref. | 228 | Ref. | Ref. | Ref. | 193 | Ref. | Ref. | Ref. | 304 | Ref. | Ref. | Ref. | 184 | Ref. | Ref. | Ref. |
| Spring (03/21-06/20) | 157 | 1.73 | (1.10, 2.72) | 0.02 | 230 | 1.02 | (0.68, 1.53) | 0.92 | 266 | 1.35 | (0.94, 1.93) | 0.11 | 261 | 1.41 | (0.94, 2.12) | 0.10 | 268 | 1.17 | (0.78, 1.75) | 0.45 | 172 | 1.04 | (0.63, 1.72) | 0.87 |
| Summer (06/21-09/22) | 152 | 0.94 | (0.60, 1.47) | 0.77 | 214 | 0.61 | (0.41, 0.90) | 0.01 | 219 | 1.52 | (1.04, 2.22) | 0.03 | 220 | 0.95 | (0.64, 1.44) | 0.84 | 197 | 0.78 | (0.52, 1.19) | 0.25 | 125 | 0.65 | (0.39, 1.09) | 0.10 |
| Fall (09/23-12/21) | 139 | 1.26 | (0.79, 1.99) | 0.33 | 200 | 0.79 | (0.53, 1.19) | 0.26 | 258 | 1.20 | (0.84, 1.72) | 0.32 | 223 | 1.13 | (0.75, 1.71) | 0.56 | 280 | 0.83 | (0.57, 1.22) | 0.34 | 172 | 0.89 | (0.55, 1.45) | 0.63 |
| Day of the week: |  |  |  |  |  |  |  |  |  |  |  |  |  |  |  |  |  |  |  |  |  |  |  |  |
| Weekday | 572 | Ref. | Ref. | Ref. | 805 | Ref. | Ref. | Ref. | 934 | Ref. | Ref. | Ref. | 849 | Ref. | Ref. | Ref. | 987 | Ref. | Ref. | Ref. | 606 | Ref. | Ref. | Ref. |
| Weekend | 29 | 1.70 | (0.78, 3.72) | 0.19 | 50 | 1.46 | (0.78, 2.76) | 0.24 | 37 | 1.39 | (0.69, 2.80) | 0.36 | 48 | 1.20 | (0.63, 2.31) | 0.58 | 62 | 1.86 | (0.90, 3.83) | 0.09 | 47 | 0.85 | (0.44, 1.66) | 0.64 |
| No. syndrome-positive visits billed by the study physician in the previous 30 days, for the same syndrome as the verified visit (per 10 visits) | 601 | 1.03 | (0.98, 1.09) | 0.23 | 855 | 1.04 | (0.87, 1.25) | 0.67 | 971 | 0.78 | (0.54, 1.12) | 0.17 | 897 | 0.96 | (0.80, 1.15) | 0.66 | 1,049 | 1.06 | (1.02, 1.10) | <0.01 | 653 | 1.03 | (0.99, 1.07) | 0.13 |
| Workload: no. claims billed that day (per 10 claims) | 601 | 0.92 | (0.84, 1.01) | 0.08 | 855 | 0.99 | (0.92, 1.07) | 0.84 | 971 | 0.97 | (0.90, 1.04) | 0.41 | 897 | 0.92 | (0.85, 0.99) | 0.03 | 1,049 | 0.95 | (0.89, 1.02) | 0.15 | 653 | 0.98 | (0.90, 1.07) | 0.63 |

**Table 4. Billing characteristics associated with accuracy of syndrome definitions based on physician claims: results from *bivariate* regression analyses for each syndrome individually** (OR>1.00 means the billing characteristic increased the PPV of the syndrome definition, OR<1.00 means the billing characteristic reduced the PPV)

|  | **Fever syndrome**  **(N=601 visits)** | | | | **Gastrointestinal syndrome**  **(N=855 visits)** | | | | **Neurological syndrome**  **(N=971 visits)** | | | | **Rash syndrome**  **(N=897 visits)** | | | | **Respiratory syndrome**  **(N=1,049 visits)** | | | | **Influenza-like illness**  **(N=653 visits)** | | | |
| --- | --- | --- | --- | --- | --- | --- | --- | --- | --- | --- | --- | --- | --- | --- | --- | --- | --- | --- | --- | --- | --- | --- | --- | --- |
| **Billing practices** | **No. visits** | **OR** | **95% CI** | **P Value** | **No. visits** | **OR** | **95% CI** | **P value** | **No. visits** | **OR** | **95% CI** | **P value** | **No. visits** | **OR** | **95% CI** | **P value** | **No. visits** | **OR** | **95% CI** | **P value** | **No. visits** | **OR** | **95% CI** | **P value** |
| Who entered the diagnostic code in the claim? |  |  |  |  |  |  |  |  |  |  |  |  |  |  |  |  |  |  |  |  |  |  |  |  |
| Physician | 95 | Ref. | Ref. | Ref. | 133 | Ref. | Ref. | Ref. | 137 | Ref. | Ref. | Ref. | 130 | Ref. | Ref. | Ref. | 158 | Ref. | Ref. | Ref. | 116 | Ref. | Ref. | Ref. |
| Secretary or nurse | 407 | 1.51 | (0.96, 2.37) | 0.07 | 571 | 1.21 | (0.82, 1.78) | 0.33 | 659 | 1.17 | (0.80, 1.70) | 0.42 | 614 | 0.78 | (0.51, 1.20) | 0.26 | 702 | 0.75 | (0.48, 1.15) | 0.18 | 431 | 1.11 | (0.69, 1.78) | 0.66 |
| Off-site billing company or  RAMQ (i.e., paper billing) | 99 | 1.34 | (0.76, 2.35) | 0.31 | 151 | 1.56 | (0.96, 2.55) | 0.08 | 175 | 0.87 | (0.55, 1.36) | 0.53 | 153 | 0.73 | (0.44, 1.22) | 0.23 | 189 | 0.69 | (0.41, 1.15) | 0.16 | 106 | 0.93 | (0.51, 1.68) | 0.80 |
| Billing software used: |  |  |  |  |  |  |  |  |  |  |  |  |  |  |  |  |  |  |  |  |  |  |  |  |
| Soft Informatique | 126 | Ref. | Ref. | Ref. | 217 | Ref. | Ref. | Ref. | 241 | Ref. | Ref. | Ref. | 216 | Ref. | Ref. | Ref. | 266 | Ref. | Ref. | Ref. | 132 | Ref. | Ref. | Ref. |
| Purkinje | 135 | 1.21 | (0.74, 1.97) | 0.45 | 197 | 0.81 | (0.54, 1.22) | 0.31 | 222 | 1.93 | (1.30, 2.85) | 0.001 | 206 | 1.10 | (0.72, 1.67) | 0.67 | 233 | 1.67 | (1.11, 2.53) | 0.02 | 139 | 1.65 | (0.95, 2.87) | 0.08 |
| ADN Medical | 80 | 0.97 | (0.56, 1.71) | 0.92 | 100 | 1.04 | (0.62, 1.75) | 0.88 | 128 | 1.05 | (0.68, 1.63) | 0.82 | 122 | 1.19 | (0.73, 1.94) | 0.50 | 144 | 1.83 | (1.12, 3.00) | 0.02 | 105 | 1.20 | (0.68, 2.11) | 0.54 |
| Omni-Med.com Caduceus | 55 | 0.98 | (0.52, 1.85) | 0.96 | 70 | 1.04 | (0.58, 1.87) | 0.89 | 82 | 0.66 | (0.40, 1.10) | 0.11 | 80 | 1.03 | (0.59, 1.80) | 0.93 | 91 | 1.25 | (0.73, 2.15) | 0.42 | 47 | 1.84 | (0.81, 4.15) | 0.14 |
| Medicus MED-WIN | 27 | 0.95 | (0.41, 2.18) | 0.90 | 41 | 0.52 | (0.26, 1.02) | 0.06 | 43 | 0.67 | (0.35, 1.28) | 0.22 | 37 | 0.65 | (0.32, 1.32) | 0.23 | 43 | 1.39 | (0.66, 2.97) | 0.39 | 38 | 1.22 | (0.54, 2.74) | 0.63 |
| Facturation.net | 24 | 0.36 | (0.14, 0.94) | 0.04 | 25 | 0.35 | (0.15, 0.81) | 0.01 | 29 | 0.43 | (0.19, 0.94) | 0.03 | 32 | 0.84 | (0.38, 1.84) | 0.66 | 28 | 1.27 | (0.52, 2.10) | 0.60 | 19 | 0.48 | (0.18, 1.28) | 0.14 |
| ANDX Xclaim | 21 | 0.54 | (0.21, 1.40) | 0.21 | 21 | 0.73 | (0.29, 1.83) | 0.50 | 22 | 0.70 | (0.29, 1.67) | 0.42 | 17 | 0.39 | (0.15, 1.06) | 0.06 | 25 | 2.22 | (0.74, 6.67) | 0.16 | 16 | 1.88 | (0.51, 6.98) | 0.34 |
| CareOffice | 20 | 0.72 | (0.28, 1.86) | 0.50 | 21 | 0.89 | (0.35, 2.31) | 0.82 | 23 | 1.31 | (0.53, 3.20) | 0.56 | 26 | 1.85 | (0.67, 5.11) | 0.24 | 28 | 1.06 | (0.45, 2.50) | 0.90 | 26 | 1.18 | (0.46, 3.03) | 0.73 |
| Médifiche | 14 | 3.23 | (0.86, 12.13) | 0.08 | 19 | 0.77 | (0.29, 2.03) | 0.59 | 24 | 0.98 | (0.42, 2.29) | 0.96 | 19 | 0.61 | (0.23, 1.57) | 0.30 | 29 | 2.64 | (0.89, 7.84) | 0.08 | 22 | 2.75 | (0.77, 9.83) | 0.12 |
| Toubib | 13 | 0.39 | (0.12, 1.38) | 0.13 | 21 | 0.60 | (0.24, 1.48) | 0.26 | 20 | 0.70 | (0.28, 1.74) | 0.44 | 18 | 0.35 | (0.13, 0.93) | 0.04 | 23 | 0.79 | (0.32, 1.94) | 0.61 | 11 | 4.34 | (0.54, 35.06) | 0.17 |
| Other^2^ or unknown | 86 | 1.35 | (0.77, 2.35) | 0.29 | 123 | 0.57 | (0.36, 0.90) | 0.02 | 137 | 1.01 | (0.66, 1.55) | 0.97 | 124 | 1.12 | (0.69, 1.82) | 0.65 | 139 | 1.60 | (0.99, 2.61) | 0.06 | 98 | 1.42 | (0.78, 2.58) | 0.25 |
| Annual billing volume (per 1,000 claims)^1^ | 601 | 0.98 | (0.92, 1.04) | 0.44 | 855 | 0.97 | (0.92, 1.02) | 0.21 | 971 | 1.01 | (0.96, 1.06) | 0.85 | 897 | 1.02 | (0.97, 1.08) | 0.48 | 1,049 | 1.01 | (0.96, 1.07) | 0.63 | 653 | 1.03 | (0.97, 1.10) | 0.36 |
| Percent of visits with a missing or unspecified diagnostic code^1^ | 601 | 1.01 | (0.98, 1.04) | 0.39 | 855 | 0.99 | (0.97, 1.02) | 0.55 | 971 | 0.98 | (0.96, 1.01) | 0.13 | 897 | 1.02 | (0.99, 1.05) | 0.29 | 1,049 | 1.02 | (0.99, 1.06) | 0.17 | 653 | 1.00 | (0.97, 1.03) | 0.97 |
| No. distinct diagnostic codes used (per 100 codes)^1^ | 601 | 0.99 | (0.84, 1.17) | 0.93 | 855 | 1.01 | (0.87, 1.18) | 0.90 | 971 | 0.94 | (0.81, 1.08) | 0.35 | 897 | 1.03 | (0.88, 1.20) | 0.74 | 1,049 | 1.06 | (0.91, 1.24) | 0.47 | 653 | 1.38 | (1.12, 1.70) | <0.01 |

^1^ In the study year when the visit took place. The study spanned 2 years: October 1, 2005 to September 30, 2006, and October 1, 2006 to September 30, 2007.

^2^ The remaining billing softwares were aggregated under “other” because their numbers were too small to report individually in this analysis.
